# A framework to unlock marine bird energetics

**DOI:** 10.1242/jeb.246754

**Published:** 2023-12-18

**Authors:** Ruth E. Dunn, James Duckworth, Jonathan A. Green

**Affiliations:** ^1^ Lancaster Environment Centre, Lancaster University, Lancaster, Lancashire, LA1 4YQ, UK; ^2^ The Lyell Centre, Heriot-Watt University, Edinburgh, Lothian, EH14 4BA, UK; ^3^ School of Environmental Sciences, University of Liverpool, Liverpool, Merseyside, L3 5DA, UK

**Keywords:** Energy expenditure, Field metabolic rate, Seabirds, Sea ducks, Time budget, Great auk

## Abstract

Energetics can provide novel insights into the roles of animals, but employing an energetics approach has traditionally required extensive empirical physiological data on the focal species, something that can be challenging for those that inhabit marine environments. There is therefore a demand for a framework through which to estimate energy expenditure from readily available data. We present the energetic costs associated with important time- and energy-intensive behaviours across nine families of marine bird (including seabirds, ducks, divers and grebes) and nine ecological guilds. We demonstrate a worked example, calculating the year-round energetic expenditure of the great auk, *Pinguinus impennis*, under three migration scenarios, thereby illustrating the capacity of this approach to make predictions for data-deficient species. We provide a comprehensive framework through which to model marine bird energetics and demonstrate the power of this approach to provide novel, quantitative insights into the influence of marine birds within their ecosystems.

## Teaser

**Summary:** An approach through which to integrate activity-specific energetic costs with time–budget data and morphological information to estimate the energy expenditure of any marine bird population.

## INTRODUCTION

The transfer and storage of energy and nutrients is fundamental for global ecosystem functioning (Bauer and Hoye, 2014). Specifically, predators and other consumers play a critical role in the functioning of ecosystems as they transfer energy between trophic levels. Furthermore, their large bodies and high mobility mean that they can act as a vector for the movement of energy and nutrients within habitats and across ecosystem boundaries (Schmitz et al., 2010). The role of wide-ranging consumers is particularly key in marine ecosystems because of their inherent ‘openness’ and the resultant potential for inter-ecosystem linkages (McCauley et al., 2012), yet, paradoxically, these species can be the hardest to study. Indeed, investigating the foraging behaviour, movement ecology and physiology of secretive, wild, marine species is challenging and integrating these elements to calculate energy budgets has only been possible for a small number of shark, whale and pinniped species to date (Bejder et al., 2019; Lowe, 2002; Sparling et al., 2008). However, the reliance of avifauna on terrestrial breeding grounds means that marine birds (seabirds, ducks, divers and grebes) are relatively accessible, at least for a period of the annual cycle, allowing enhanced insights into their physiology and ecology (Bernard et al., 2021). Indeed, by attaching biologging devices to these animals during their breeding seasons, we now know more than ever about their behaviour, ecology and physiology, whilst they are out of sight within their marine habitats (Croxall et al., 2005; Egevang et al., 2010).

To fuel their energetic requirements, marine birds have evolved to prey on a range of fish and invertebrate species. They adeptly occupy a multitude of foraging niches that range from the capture of flying fish (*Exocoetidae* spp.) from the surface of tropical waters (Lerma et al., 2020), to the retrieval of fishes and squid from depths of over 500 m (Wienecke and Robertson, 1997). In total, marine birds extract almost 100 million tonnes of food each year from a variety of marine habitats, with consequences for ecosystem management (Karpouzi et al., 2007). Indeed, there is demand for energetic studies to provide applied insights into a range of topical issues including potential conflicts with fishing industries (Danckwerts et al., 2014), alterations to governance on discards (Sherley et al., 2020), and the displacement effects of marine developments (Croll et al., 2022). However, despite their relative ease of study in comparison to other marine consumers, our knowledge of the energetics of marine birds remains patchy, varying temporally, spatially and interspecifically.

The energy expenditure of some species of marine bird has been measured using methods such as accelerometery, doubly labelled water and the heart rate method (Green, 2011; Green et al., 2009; Shaffer, 2011). For species where such measurements have not yet been made, we can estimate basal metabolic rate (BMR) via allometric scaling relationships (Ellis and Gabrielsen, 2002). Similarly, estimations of the field metabolic rate (FMR; the sum of energy that a wild animal metabolises over a specified period of time) of breeding seabird species are now increasingly common, thereby allowing inferences into the energetic expenditure and requirements of this group of predators (Dunn et al., 2018). To extrapolate beyond the confines of the breeding seasons of marine birds, year-round biologging devices have been used to determine behavioural time–activity budgets and increasingly also year-round energy budgets, in recognition of the major role of behaviour-specific energetic costs in driving energy budgets (Brown et al., 2023; Buckingham et al., 2023; Burke et al., 2015; Dunn et al., 2020; Elliott and Gaston, 2014). Currently, however, these inferences remain difficult for species where accelerometery, doubly labelled water and heart rate data from the breeding season are not available to aid the interpretation of year-round data. To help unlock the field of energetics for all marine bird researchers, we set out to draw upon the existing knowledge base and present an approach that captures the main energetic costs, enabling the estimation of the daily energy expenditure of any marine bird population at any period in the annual cycle for which time–budget data can be measured or estimated.

## MATERIALS AND METHODS

### Compiling marine bird BMR multipliers

**Fig. 1.**
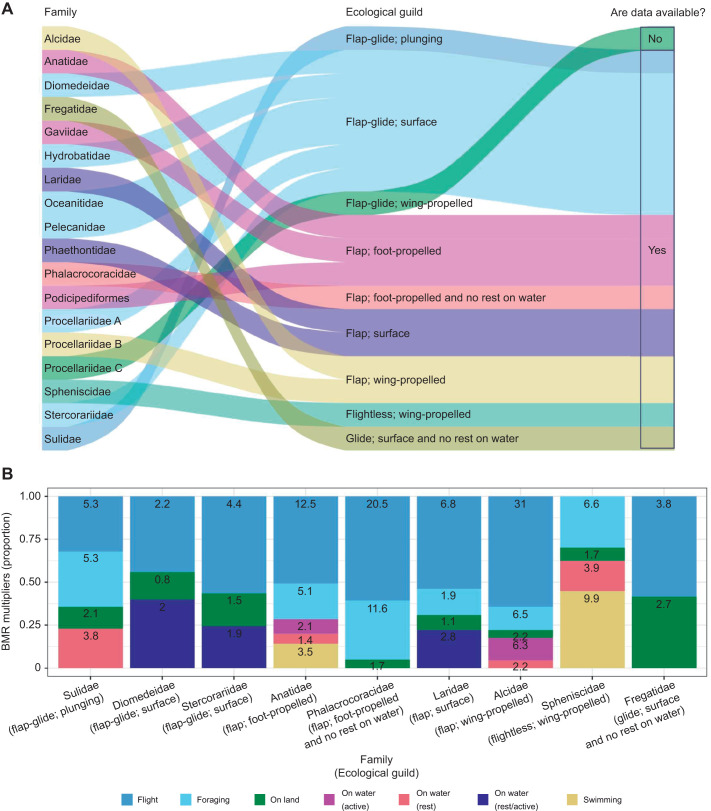
**The ecological guilds and basal metabolic rate (BMR) multipliers of marine bird families.** (A) The nine marine bird ecological guilds and whether BMR multiplier data are available for them. There are 16 families of marine birds and the relatively speciose Procellariidae have been split into three classifications according to the variation in their foraging and flight behaviour: Procellariidae A (fulmarine petrels, gadfly petrels and prions), B (diving petrels) and C (shearwaters). The ecological guilds are based on predominant flight style (flap, glide, flap-glide or flightless), foraging style (plunge diving, surface feeding, wing-propelled diving or foot-propelled diving), and whether the bird rests on land and/or on water (or not). (B) The activity-specific BMR multipliers for nine families of marine birds represented as within-family proportions, with the value of each activity- and family-specific BMR multiplier labelled.

We assigned each family of marine bird, as defined by BirdLife International 2023 (www.datazone.birdlife.org/species/search), to one of nine ecological guilds based on expert opinion (R.E.D., J.D. and J.A.G.) of their flight, foraging and resting ecologies (Fig. 1A). We split the relatively speciose Procellariidae into three groups according to intra-family differences in their foraging and flight behaviour: Procellariidae A (flap-gliding surface feeders such as fulmarine petrels, gadfly petrels and prions), B (flapping wing-propelled divers such as diving petrels) and C (flap-gliding wing-propelled divers such as shearwaters).

Between November 2022 and March 2023 inclusive, we performed a search of the peer-reviewed literature available on Google Scholar for studies of marine bird energetics. Searches were conducted for three groups of search terms: (1) ‘energ*’, ‘metabol*’ and ‘duck’, (2) ‘metabol*’ and ‘seabird*’, and (3) ‘energ*’ and ‘seabird*’. We restricted our search to 369 species of marine bird, as defined above, omitting the Scolopacidae family (sandpipers) from our analyses because of physiological differences and a general lack of marine dependence in comparison to the other families within the Charadriiformes order, which are also excluded from BirdLife International's list. When looking for values for specific families, we also conducted additional specific searches outside the pre-defined search terms.

We scanned all resultant abstracts for an indication that publications reported estimates of the costs associated with performing different activities and, if appropriate, the full text was skimmed. BMR multiplier values were then either extracted or calculated from the data presented and BMR values cited within the containing text. Where multiple values were available for one family of marine bird, we used an expert judgement approach where we either extracted the activity-specific BMR multiplier that was most up to date, or which we were most confident in, whereby it best reflected free-ranging behaviour, or minimised the number of different sources being used, thereby increasing comparability between activities. Where activity-specific costs were provided as multiples of resting metabolic rate (RMR), we converted RMR values into multipliers of BMR (by dividing the RMR by the BMR multiplier that we had for resting behaviour) to then assist us in also converting the activity-specific costs into multiples of BMR as well. We evaluated which marine bird ecological guilds we had BMR multipliers for. Where values for an ecological guild did not exist, we provide a suggestion of which activity-specific values from other ecological guilds might be most appropriate to use, based on phylogenetic and behavioural similarities.

### Calculating marine bird daily energy expenditure

An animal's external environment and how it allocates time between different costs drives its energetic expenditure (Ricklefs and Wikelski, 2002). To calculate the energy expenditure of any species of marine bird, we therefore followed an established approach whereby the time spent per day in pre-defined activities is multiplied by the species' BMR and an appropriate activity-specific BMR multiplier (Dunn et al., 2020). These values are then summed to give a daily energy expenditure.

If species-specific values for BMR from empirical data are not available, family-specific estimates for a number of seabird families are provided within Ellis and Gabrielsen (2002) and should be used where possible. Alternatively, allometric scaling equations can be used to parameterise the unknown physiological metrics that influence a wild animal's energy budget: BMR, lower critical temperature and thermal conductance. Initially, based on an individual's body mass (g), we can calculate its BMR (kJ h^−1^). This could be a single estimate for the year, or a range of values incorporating temporal variation if this is known. BMR can be calculated as the average of two relationships (Eqns 1A and 1B) previously presented for seabirds and ducks (Ellis and Gabrielsen, 2002; McKinney and McWilliams, 2005):
(1rmA)

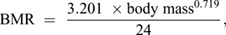

(1rmB)

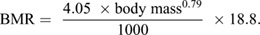


When selecting which activity-specific BMR multipliers to use, those obtained for the ecological guild adopted by the species' family should be considered (Fig. 1). When a full set of activity-specific BMR multipliers are not available for a particular ecological guild, the most appropriate activity-specific BMR multipliers, determined via the species' ecology and physiology, should be selected, possibly from across multiple ecological guilds.

When data on the environmental temperatures experienced by an individual are available, we can also incorporate activity-specific thermoregulatory energetic costs within our energy budget calculations to improve estimates, particularly for time periods when birds are inactive. To estimate the these, we must first calculate the individual's lower critical temperature (LCT; °C), the temperature below which an additional cost of thermoregulation is incurred (Kendeigh et al., 1977):
(2)

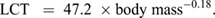


Using equations previously presented for aquatic birds, we can also calculate the individual's thermal conductance (TC; kJ h^−1^ °C^−1^ kg^−1^) when submerged in water, sitting on water, or in air (De Vries and Van Eerden, 1995):
(3rmA)

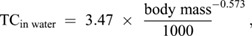

(3rmB)

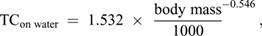

(3rmC)

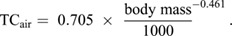


We can then correspond these thermal conductance values to the activities performed by different species of marine bird. For example, the TC_in_ _water_ value might be used for species that swim and forage with their entire bodies beneath the water surface, TC_on_ _water_ might be used for species that spend time either resting or being active on the surface of the water, and TC_air_ might be used for species that spend time on land. We assume that when in flight, marine birds use active flapping flight producing excess heat that compensates for any thermoregulatory requirements (Schraft et al., 2019), and/or live in tropical habitats, and/or have other adaptations, such as darker wings, which compensate for the costs of thermoregulation and help maintain thermoneutrality (Rogalla et al., 2021). Furthermore, we exclude other less consequential sources of energy expenditure such as digestion, growth, reproduction, moult and variation in resting costs.

When environmental temperatures (sea surface temperature or the temperature of air, depending on the activity) fall below an individual's LCT during time spent (h) in relevant activities, the energetic costs of thermoregulation (kJ) can be calculated as follows:
(4)

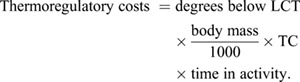


Ultimately, marine bird energy expenditure can be calculated as the sum of activity-specific energetic costs and thermoregulatory costs.

### Year-round energy expenditure by the great auk

To demonstrate the capacity of our approach to address ecological concepts, we calculated the year-round daily energy expenditure of a great auk, *Pinguinus impennis* (Linnaeus, 1758), a species made extinct in 1884 (Bengtson, 1984). Initially, based on year-round empirical data from a closely related extant species (common guillemot, *Uria aalge*), we constructed theoretical year-round activity budgets and extracted environmental temperature data based on three theoretical migration scenarios: (A) the great auk stayed close to its breeding colony throughout the year and returned to land during the night (similar to populations of gentoo penguin, *Pygoscelis papua*; Tanton et al., 2004), (B) the great auk stayed close to its breeding colony throughout the year and stayed at sea during the night when not under the constraints of the breeding season (similar to populations of common guillemot; Harris et al., 2015), (C) outside the breeding season, the great auk undertook large migratory journeys to distinct wintering grounds (similar to populations of Atlantic puffins, *Fratercula arctica*; Fayet et al., 2016). We created a set of simulations, detailed in the Supplementary Materials and Methods, and, given the ecology and physiology of great auks (Bengtson, 1984), we derived activity-specific costs from values obtained for a wing-propelled, non-flying bird that does rest on water (Fig. 1B and Table 1). Ultimately, we were able to combine these input data and activity-specific BMR multipliers to simulate the activity budget and corresponding variation in energetic expenditure of a representative individual great auk under three migration scenarios.

**Table 1.**
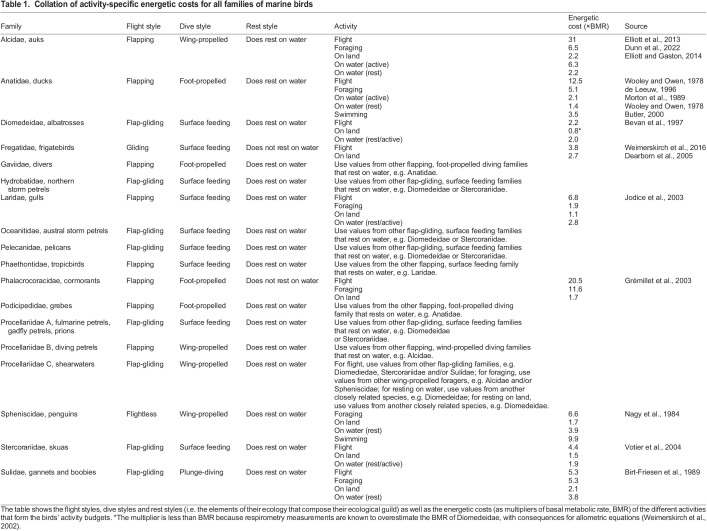
Collation of activity-specific energetic costs for all families of marine birds

## RESULTS AND DISCUSSION

We obtained activity-specific BMR multipliers from nine families of marine bird: Alcidae, Anatidae, Laridae, Fregatidae, Phalacrocoracidae, Diomedeidae, Spheniscidae, Stercorariidae and Sulidae (Table 1). The foraging, flight and resting behaviours of these nine families encompassed eight of the nine ecological guilds that we had grouped the families into: flap-gliding plunge divers, flap-gliding surface feeders, flapping foot-propelled divers, flapping foot-propelled divers that do not rest on water, flapping surface feeders, flapping wing-propelled divers, gliding surface feeders, and flightless wing-propelled divers (Fig. 1A). Another nine families, for which we were not able to find activity-specific BMR multipliers, could be assigned to one of these eight ecological guilds based on the assumption that their multipliers would be similar (Fig. 1A). We were not able to find data for flap-gliding wing-propelled divers (i.e. ‘Procellariidae C’, shearwaters) and, instead, we encourage the use of activity-specific multipliers from closely related species and those with similar ecological guilds (detailed in Table 1). For example, multipliers for flight and ‘on water’ could be borrowed from other closely related families that employ flap-gliding and that rest on water, and multipliers for foraging could be taken from other wing-propelled foragers such as the Alcidae family. Future studies that seek to quantify the activity-specific energetic costs of shearwaters would be extremely valuable with regards to closing this outstanding knowledge gap.

There was variation in the magnitude of the BMR multipliers between the different families of marine bird. Flight costs were high in comparison to the energetic costs associated with other activities across taxa (Fig. 1B). Our cross-taxa data collation confirms that flight was particularly costly for Alcidae species (31×BMR), which have wings that are evolved for optimal dive performance but are less efficient when used for flapping flight (Elliott et al., 2013). Contrastingly, Diomedeidae species have low energetic costs associated with their flight activity (2.2×BMR) in addition to their time spent on land (0.8×BMR) or on water (2×BMR) (Fig. 1B), with take-offs hypothesised to be their most expensive activity because of the requirement of continuous, costly flapping (Sakamoto et al., 2013). Flight is also costly for Phalacrocoracidae (20.5×BMR) because of their wings not being optimised solely for flight but also to reduce drag and buoyancy during underwater foraging (Stothart et al., 2016).

In recognition of the major role of behaviour-specific energetic costs in driving energy budgets, time–energy budgets have long been used to estimate daily energy expenditure across a range of marine bird taxa (Bunce, 2001; Fort et al., 2011; Grémillet et al., 1995; Regular et al., 2014; Votier et al., 2004), including, increasingly, outside the breeding season (Buckingham et al., 2023; Dunn et al., 2022; Elliott and Gaston, 2014; Grémillet et al., 2005). Our framework expands on these single species studies and the activity-specific BMR multipliers compiled in Fig. 1B can be combined with BMR estimates, empirical activity budget data and, where available, thermal conductance estimates and environmental temperature data to estimate the daily energy expenditure of any population of marine bird. Like any similar time–energy budget study, estimates are dependent on and sensitive to the values of BMR used. We demonstrate how to estimate BMR from published body mass alone, but any further information on species-, population- or individual-specific measures of body mass can be used within Eqns 1A and 1B to improve estimates of BMR. Even better would be to use taxon- or species-specific BMR measurements or relationships between mass and BMR (e.g. Ellis and Gabrielsen, 2002) and any known influences on BMR, as this can also vary as a result of ecological effects, temporal variation and measurement differences (McKechnie and Wolf, 2004). Other species-specific insights might include known relationships between mass and thermal conductance, or other known aspects of activity-specific rates of energy expenditure. Further data streams that would improve estimates include knowledge of depth- and duration-related variation in the energetic costs of diving (as in Elliott et al., 2013; Knower Stockard et al., 2005). Furthermore, birds might expend different amounts of energy whilst ‘on land’ as a result of the differing costs associated with egg incubation, brooding young chicks and then rearing them (Green et al., 2013), or being engaged in different behaviours such as resting, preening, walking or wing stretching (Grémillet et al., 1995). Additionally, our findings reveal some scope for variation in activity costs within ecological guilds, and we therefore encourage future studies to use species-specific BMR multipliers where they are available, and also to generate new multipliers to both unlock insights into individual species and improve this approach overall. This being said, the framework that we outline here allows the incorporation of nuances via the optional addition of location-specific thermoregulatory costs when environmental temperature data (usually air and sea surface) are available (Eqns 2 and 3). In this way, informed estimates of daily energy expenditure can be generated for marine birds at any point throughout the annual cycle where activity budget data are available.

**Fig. 2.**
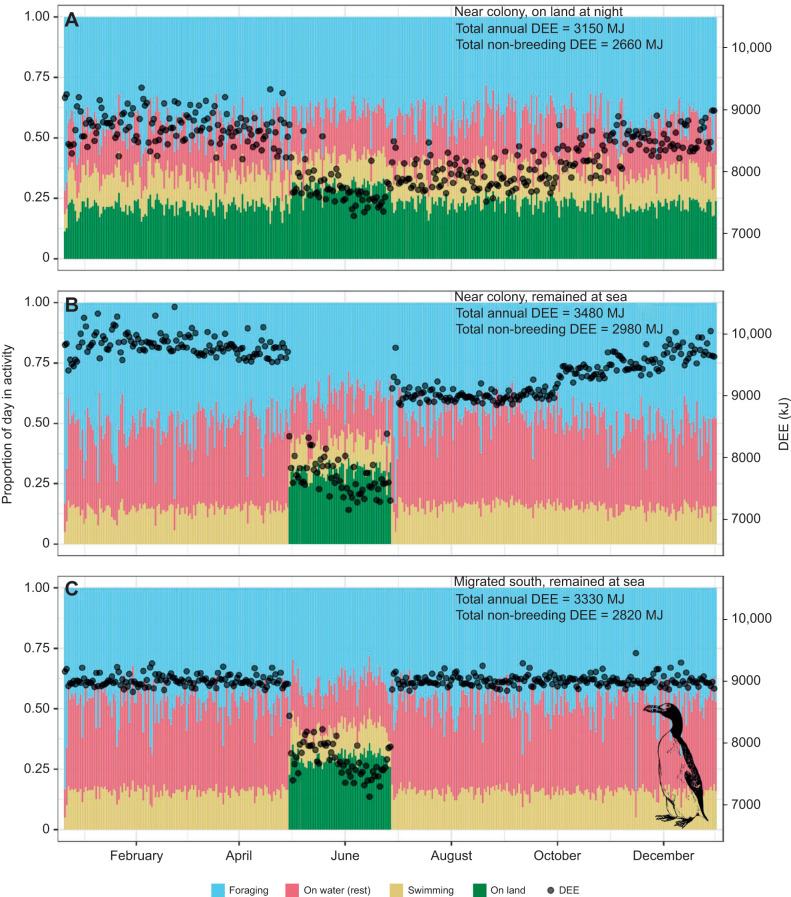
**A theoretical reproduction of the daily energy expenditure of a great auk, *Pinguinus impennis*, through an annual cycle under three migration scenarios.** (A) The bird stayed close to its breeding colony throughout the year and returned to land during the night. (B) The bird stayed close to its breeding colony throughout the year and stayed at sea during the night when not under the constraints of the breeding season. (C) Outside the breeding season, the bird undertook large migratory journeys to distinct wintering grounds. Daily energy expenditure (DEE) estimates (circles) were calculated based on theoretical behavioural budgets (coloured stacked bars) and estimates of foraging efficiency, combined with activity-specific BMR multipliers and theoretical BMR and thermoregulatory costs derived from allometric scaling equations. Great auk image ^©^Openclipart.

To demonstrate the flexibility and potential of our approach for answering broad-scale questions about animal energetics, we provide a case study in which we estimated the year-round daily energy expenditure of the great auk, a marine bird species that lacks comprehensive physiological information and empirical data because it was hunted to extinction in 1884 (Bengtson, 1984). We were able to reconstruct energy budgets under three plausible migration/non-breeding scenarios (based on strategies employed by extant species with similar lifestyles) considering temporal changes in activity budgets, and both spatial and temporal variation in temperature. We showed that if, during the non-breeding season, the great auk made diurnal trips to sea to forage and returned to land during the night (similar to South Georgian gentoo penguins; Tanton et al., 2004), it would have expended approximately 12% less energy than if it had stayed at sea throughout the entire non-breeding period (Fig. 2). Furthermore, remaining within cool Arctic oceans would have incurred an approximate 5% increase in energy expenditure throughout the non-breeding period relative to that expended if it had adopted a southern migration to the Atlantic Ocean near Morocco (the southernmost location that great auk bones have been found; Campmas et al., 2010), driven by cooler sea surface temperatures and consequent increased thermoregulatory costs (Dunn et al., 2020). Although the breeding season is typically an expensive period for marine birds, with high associated energetic costs (Dunn et al., 2018), we did not account for the intrinsic costs of reproduction (including egg development and chick provisioning) within our simulation and therefore observed comparatively low energetic costs associated with colony attendance during May and June (Fig. 2). This being said, the patterns in daily energy expenditure that we observed are not dissimilar to those observed across multiple colonies of common guillemot, an extant species that is one of the great auk's closest relatives (Buckingham et al., 2023). During this period, assuming an assimilation efficiency of 74.4% (as in Brünnich's guillemot, *Uria lomvia*; Brekke and Gabrielsen, 1994), the great auk would have had to consume 5790±248 kJ day^−1^ of prey in order to maintain its body mass, or 36±2 sand lance *Ammodytes dubius* (assuming the energy content of a 20 cm sand lance was 158 kJ; Bowen et al., 2002). Our ability to reconstruct the energy expenditure of the great auk, a hugely data-deficient species, and investigate intra-annual variability in its energetics demonstrates both the power and flexibility of our approach, as well as its utility in exploring novel ecological scenarios and concepts.

Here, we have outlined an approach that can be used to estimate the daily energy expenditure of marine bird species at any time throughout the annual cycle with varying degrees of accuracy depending on the availability of empirical data. While subtleties such as small changes in body mass or time-limited activities such as reproduction might not be captured, this approach still enables us to evaluate patterns in energy expenditure and answer comparative questions. We advocate that this approach is employed to help quantify the influence marine birds exert on the ecosystems they inhabit, in addition to how they might be affected by anthropogenic impacts. For example, studying marine bird energetics has allowed the quantification of the prey that seabirds might consume (Brooke, 2004; Cury et al., 2011) as well as the quantities of nutrients that they then transfer from their marine foraging habitats to their terrestrial breeding sites and adjacent nearshore systems (Graham et al., 2018). Our approach also provides a timely tool through which to quantify the impacts of emerging catalysts for changes to marine bird activity budgets, energetics and consequent demographics, such as fisheries interactions (Searle et al., 2023), wind farm developments (Masden et al., 2010), increased storminess (Fort et al., 2009) and temperature changes (Oswald and Arnold, 2012). Indeed, quantifying the energetics of an individual bird can allow the development of insights into population dynamics, as individuals must balance their energetic budgets to ensure survival (Tomlinson et al., 2014), as well as provide answers to questions regarding evolutionary theory (Ballance et al., 2009). Furthermore, the framework presented here may also set a precedent for the creation and use of standardised workflows for the study of other taxa.

## Supplementary Material

10.1242/jexbio.246754_sup1 Supplementary information

## ECR Spotlight

This article has an associated ECR Spotlight interview with Ruth Dunn and James Duckworth.
