# Supplementary material for: A framework to unlock marine bird energetics
Source: J Exp Biol. 2023 Dec 18;226(24):jeb246754. doi: 10.1242/jeb.246754 (PMC10753490; doi:10.1242/jeb.246754)
Supplement: Supplementary information [file jexbio-226-246754-s1.pdf]

Supplementary Materials and Methods

We reconstructed theoretical year-round activity budgets of a great auk based on three contrasting migratory strategies: A) the bird stayed close to their breeding colony throughout the year and returned to land during the night (similar to the gentoo penguin *Pygoscelis papua*), B) the bird stayed close to their breeding colony throughout the year and stayed at-sea during the night when not under the constraints of the breeding season (similar to the common guillemot *Uria aalge*), C) outside the breeding season, the bird undertook large migratory journeys to distinct wintering grounds (similar to the Atlantic puffin *Fratercula arctica*).

To generate estimations of great auk energy expenditure under these different scenarios, we created a set of three individual based simulations using R version 4.2.4 (R code available on GitHub). To do this we used the best available data on the ecology and physiology of the great auk, closely related species and species with similar lifestyles. Resulting estimates may not exactly recreate the life of the great auk but should be sufficient for the comparative exercise we present here. We simulated the body mass of an individual great auk (M, g) from a normal distribution that had a mean of 5,000 and a standard deviation of 200 (Bengtson, 1984). Foraging efficiency (i.e., the amount of energy gained per hour spent foraging, f, kJ hour^{-1}) was assumed to scale allometrically with body size and was therefore simulated from a normal distribution with a mean of 800 and a standard deviation of 100 (scaled from values from common guillemots (Dunn et al., 2022)). We used these values to simulate time spent foraging (F) at a daily scale (i):

$$F_i = \frac{\mathrm{N}\big((M_o - M_i \times T), 30\big) + \mathrm{N}\big(E_{i-1}, (E_{i-1} \times 0.05)\big)}{f} \mathrm{T}(0,20)$$

Journal of Experimental Biology • Supplementary information

Here, M_o was the individuals 'ideal' mass, T was the energetic density of body tissue (38 kJ g^{-1} (Gabrielsen, 1996)), and E_{i-1} was the energy expenditure of the individual on the previous day.

The rest of the individual's daily time activity budget (time spent on land (L), resting on water (W), and swimming (S)) was parameterised differently depending on the time of year and its migratory strategy. When the bird was breeding its activity budget was parameterised as follows:

$$L_i = (24 - F_i) \times 0.5$$

$$W_i = (24 - F_i) \times 0.3$$

$$S_i = (24 - F_i) \times 0.2$$

When the bird was not breeding, but was close to the breeding colony throughout the annual cycle and returned to land at night its activity budget was parameterised as follows:

$$L_i = (24 - F_i) \times 0.4$$

$$W_i = (24 - F_i) \times 0.35$$

$$S_i = (24 - F_i) \times 0.25$$

When the bird was not breeding and was at sea throughout the annual cycle its activity budget was parameterised as follows:

Journal of Experimental Biology • Supplementary information

$$L_i = 0$$

$$W_i = (24 - F_i) \times 0.7$$

$$S_i = (24 - F_i) \times 0.3$$

When the bird was not breeding and was commuting (we assumed a ten day outward and return commute period) its activity budget was parameterised as follows:

$$L_i = 0$$

$$W_i = (24 - F_i) \times 0.5$$

$$S_i = (24 - F_i) \times 0.5$$

We calculated the thermoregulatory costs associated with the individual's time activity budget throughout its annual cycle. To do this, we assumed that sea surface temperature and air temperature were similar and extracted daily sea surface temperature data using the R package 'rerddapXtracto' (Mendelssohn, 2022). To simulate the temperature experienced by an individual near the breeding colony, we extracted sea surface temperature data from across an annual cycle from around Eldey Island, Iceland (the last known breeding location of the great auk; 63°44′27.2″ N 22°57′27.2″ W). To simulate the temperature experienced by an individual that an undertaken a large migratory journey, we extracted sea surface temperature data from the Atlantic Ocean near Temara, Morocco (33°55′36″ N 6°54′44″ W), the furthest south that great auk bones have been found (Campmas et al., 2010).

Based on the known ecology and physiology of great auks (Bengtson, 1984), we derived activity-specific energetic costs from values obtained for a wing-propelled, non-flying bird

Journal of Experimental Biology • Supplementary information

that does rest on water (i.e., the Spheniscidae family; Table S2). We then used the workflow presented within the main text to calculate the basal metabolic rate (BMR) of the individual great auk, as well as its lower critical temperature (LCT), medium-specific thermal conductance, thermoregulatory costs. Ultimately, we were able to combine these input data and activity-specific BMR multipliers to simulate the activity budget and corresponding variation in energetic expenditure throughout the annual cycle of an individual great auk under three distinct migration scenarios.

References

Bengtson, S.-A. (1984). Breeding ecology and extinction of the great auk (*Pinguinus impennis*): Anecdotal evidence and conjectures. *Auk* **101**, 1–12.

Campmas, E., Laroulandie, V., Michel, P., Amani, F., Nespoulet, R. and Mohammed, A. E. H. (2010). A Great Auk (*Pinguinus impennis*) in North Africa: Discovery of a bone remain in Neolithic layer of El Harhoura 2 Cave (Temara, Morocco). In *Birds in Archaeology: Proceedings of the 6th Meeting of the ICAZ Bird Working Group in Groningen*, pp. 1–233. Barkhuis.

Dunn, R. E., Green, J. A., Wanless, S., Harris, M. P., Newell, M. A., Bogdanova, M. I., Horswill, C., Daunt, F. and Matthiopoulos, J. (2022). Modelling and mapping how common guillemots balance their energy budgets over a full annual cycle. *Funct. Ecol.* **36**, 1612–1626.

Gabrielsen, G. W. (1996). Energy expenditure of breeding Common Murres. *Occasional Paper of the Canadian Wildlife Service* 49–58.

Mendelssohn, R. (2022). *rerddapXtracto: Extracts Environmental Data from 'ERDDAP' Web Services. R package version 1.1.4.*

Journal of Experimental Biology • Supplementary information
